# RNA interference as a key to knockdown overexpressed cyclooxygenase-2 gene in tumour cells

**DOI:** 10.1038/sj.bjc.6603094

**Published:** 2006-04-11

**Authors:** A Strillacci, C Griffoni, E Spisni, M C Manara, V Tomasi

**Affiliations:** 1 Department of Experimental Biology, University of Bologna, via Selmi 3, Bologna 40126, Italy; 2 Center for Applied Biomedical Research (CRBA), St Orsola-Malpighi University Hospital, Bologna, Italy; 3 Laboratorio di Ricerca Oncologica, Istituti Ortopedici Rizzoli, Bologna, Italy

**Keywords:** RNA Interference, COX-2, angiogenesis, colon cancer

## Abstract

Silencing those genes that are overexpressed in cancer and contribute to the survival and progression of tumour cells is the aim of several researches. Cyclooxygenase-2 (COX-2) is one of the most intensively studied genes since it is overexpressed in most tumours, mainly in colon cancer. The use of specific COX-2 inhibitors to treat colon cancer has generated great enthusiasm. Yet, the side effects of some inhibitors emerging during long-term treatment have caused much concern. Genes silencing by RNA interference (RNAi) has led to new directions in the field of experimental oncology. In this study, we detected sequences directed against COX-2 mRNA, that potently downregulate COX-2 gene expression and inhibit phorbol 12-myristate 13-acetate-induced angiogenesis *in vitro* in a specific, nontoxic manner. Moreover, we found that the insertion of a specific cassette carrying anti-COX-2 short hairpin RNA sequence into a viral vector (pSUPER.retro) greatly increased silencing potency in a colon cancer cell line (HT29) without activating any interferon response. Phenotypically, COX-2 deficient HT29 cells showed a significant impairment of their *in vitro* malignant behaviour. Thus, the retroviral approach enhancing COX-2 knockdown, mediated by RNAi, proved to be an useful tool to better understand the role of COX-2 in colon cancer. Furthermore, the higher infection efficiency we observed in tumour cells, if compared to normal endothelial cells, may disclose the possibility to specifically treat tumour cells without impairing endothelial COX-2 activity.

The pharmacological approach to cancer treatment (chemotherapy) is often hindered by the lack of specificity and by acquired cancer cells drug resistance caused by overexpressed P-glycoprotein family proteins, which carry drugs out of cells. The advancement in the field of RNA interference (RNAi) has opened up a completely new strategy to silence genes involved in tumour progression and to downregulate genes coding for P-glycoproteins in a high specific manner (Meister and Tuschl, 2004).

The idea of silencing those genes which are overexpressed in malignant cells is, of course, not new. In the past few years, several attempts to obtain specific knockdown have been performed by using stabilised antisense oligonucleotides and ribozymes (Zang *et al*, 2004). Even though some success has been reported, failure of most of clinical trials has been attributed to the assumption that *in vivo* delivery is critical, owing to the toxicity and instability of molecules used (Mello and Conte, 2004). Several reports now indicate that these problems have been overcome by the use of RNAi (Meister and Tuschl, 2004).

RNAi is considered a form of post-transcriptional gene silencing, a phenomenon first described in plants, nematodes, protozoan, invertebrate species and only recently found also in mammalians. Observations in *Caenorhabditis elegans* disclosed the remarkable capacity of double-stranded RNA molecules (dsRNA) to specifically and potently disrupt the expression of genes containing sequences homologous to the dsRNA. The mechanism involves the inhibition of translation and the enzymatic degradation of target mRNAs. Fire *et al* (1998) argued that since few molecules of injected dsRNA were required to affect a specific mRNA expression, a catalytic amplification component was involved. The amplification process has been at least partially explained by the discovery of the dsRNA enzymatic conversion into 21–25 nt oligoribonucleotides. The 21–25 nt oligoribonucleotides, also called small interfering RNAs (siRNAs), may act on mRNA targets as antisense oligonucleotides.

Synthetic siRNAs appeared as an important research tool for understanding the function of a gene by silencing its expression when Elbashir *et al* (2001a) reported that gene expression in mammalian cell lines can be effectively silenced by transfecting cells with synthetic siRNAs. The prospect of using siRNAs as potent and specific inhibitors to any target gene provides a new therapeutical approach for many untreatable diseases, such as viral infections, neurodegenerative diseases and cancer.

At this time, it is widely accepted that in mammalians, as in other organisms, physiological silencing mechanisms are based on RNAi mediated by specific dsRNA structures, called microRNAs (miRNAs). It has been recently demonstrated that the processing of short hairpin RNA structures (shRNAs) overlaps the cellular pathways in which miRNAs are involved (Bartel, 2004). This further strengthened the concept that the use of siRNAs is physiologically relevant in diseases treatment (Bartel, 2004). It has also been proposed that the overexpression of genes, like cyclooxygenase-2 (COX-2), in cancer cells (Gupta and Dubois, 2001; Romano and Claria, 2003; Tuynman *et al*, 2004) may be due to the disruption of a control network in which relevant microRNA genes are implicated (Michael *et al*, 2003; Bartel, 2004; He *et al*, 2005; Lu *et al*, 2005).

The field of COX inhibitors has a record of continuous progress starting from the introduction of aspirin in 1898. A large group of nonsteroidal anti-inflammatory drugs (NSAIDs) inhibiting both COX-1 and COX-2 isoforms followed. Only recently selective COX-2 inhibitors (Coxibs) have been developed (Flower, 2003). Here, we highlight a new strategy to inhibit COX-2 activity, focusing on the role of COX-2 in angiogenesis and in colorectal cancer (CRC). Several studies reported the relevant role of COX-2 in tumour angiogenesis (Spisni and Tomasi, 1997; Iñiguez *et al*, 2003). It has been observed that blood vessels localised in proximity of tumours express high levels of COX-2 whereas normal vasculature does not (Leahy *et al*, 2002). In advanced CRC tissues, high COX-2 levels seem to promote cells invasion, tumour growth and metastasisation (Yamauchi *et al*, 2002). To this regard, aim of this study was to achieve a significant and specific downregulation of COX-2 expression by using RNAi mechanism. We found specific siRNAs molecules capable of blocking angiogenesis mediated by COX-2 overexpression in human umbilical rein endothelial cells (HUVEC) with a mechanism that does not induce, at low concentrations, the interferon system activation. In order to obtain a stable silencing of COX-2 gene, we also developed a retroviral vector-based approach, failing to infect HUVE cells, but capable of infecting to a high degree two human colorectal cancer cell lines (HT29 and HCA7). The constitutive expression of endogenous anti-COX-2 shRNAs did not trigger any interferon system response. However, the specific and long-lasting knockdown of COX-2 in HT29 cells strongly affected their *in vitro* migratory ability and their anchorage-independent growth in soft agar. Thus, this anti-COX-2 expressing vector may be a valuable tool to control the role of COX-2 expression in cancer (particularly in CRC) opening the way to specific trials *in vivo*.

## MATERIALS AND METHODS

### Cell lines

Human endothelial cells were isolated from freshly collected umbilical cords. Cells were grown in M199 medium supplemented with 20% foetal calf serum (FCS), 100 mg ml^−1^ ECGS, 100 mg ml^−1^ heparin, 2 mM L-glutamine and antibiotics (penicillin 100 U ml^−1^ and streptomycin 100 mg ml^−1^), as previously described (Spisni *et al*, 1995). Cells were maintained at 37°C in 5% CO_2_ and used for experiments between third and fifth passage. HT29 human colon cancer cells were obtained from American Type Culture Collection (Manassas, VA, USA). HCA7 cells were obtained from European Collection of Cell Cultures (ECACC; Salisbury, Wiltshire, UK). Both HT29 and HCA7 cells were cultured at 37°C in 5% CO2 in Dulbecco's modified Eagle's medium (DMEM) supplemented with 10% heat-inactivated FCS, 2 mM L-glutamine, penicillin 100 U ml^−1^ and streptomycin 100 mg ml^−1^. Foetal calf serum and DMEM were purchased from Cambrex Biowittaker, USA. All the other reagents were purchased from Sigma (Sigma Chemical, St Louis, USA).

### Synthetic siRNAs design

Four different siRNAs against COX-2 mRNA were designed as suggested by Elbashir *et al* (2002) and chemically synthesised (PROLIGO Primers and Probes, USA). The identified target sequences on COX-2 mRNA (NM000963) were respectively: bases 290–310 (siRNA-A, 5′aaactgctcaacaccggaatt3′), bases 291–311 (siRNA-B, 5′aactgctcaacaccggaattt3′), bases 1020–1040 (siRNA-C, 5′aacagagtatgcgatgtgctt3′) and bases 1429–1449 (siRNA-D, 3′aagtatcacaggcttccattg5′). We also used a scrambled siRNA (Ambion, USA) as a negative control, with no significant homology to any known gene sequences in human, mouse and rat genome. All siRNA sequences were controlled for their specificity by using BLAST database and did not show any homology to other human gene.

### siRNAs transfection

Human endothelial cells were seeded in 25 cm^2^ flasks (150 000 cells flask^−1^) and grown up to 50% confluence. After 24 h, cells were transfected with siRNAs by using Oligofectamine reagent (Invitrogen, USA) according to the manufacturer's instructions. Briefly, each siRNA (400 fmoles) was diluted in 350 *μ*l of serum-free medium. For each flask, 10 *μ*l of Oligofectamine reagent was incubated with 40 *μ*l of serum-free medium for 10 min at room temperature. Oligofectamine solution (50 *μ*l) was added to diluted siRNAs. After incubation for 20 min at room temperature, siRNAs–Oligofectamine complexes were added to flasks containing 1.6 ml of serum-free medium, resulting in 2 ml of total transfection volume and in a siRNA final concentration of 200 pM. After 4 h of incubation at 37°C, 3 ml of complete medium containing FCS and phorbol 12-myristate 13-acetate (PMA) was added (final concentration: 10% serum and 40 nM PMA).

### Western blot

Cells were scraped and lysed in lysis buffer (50 mM Tris-HCl, pH 7.5, 2 mM EDTA, 100 mM NaCl, 1% Triton X-100 and protease inhibitors mixture), additionated with 5 mM NaF, 1 mM Na_3_VO_4_, 10 mM *β*-glycerolphosphate, for the phospho-STAT-1 immunoblotting experiments. Cell lysates were incubated 1 h on ice and centrifuged at 12 000 **g** to collect supernatants. Protein concentration in supernatants was evaluated by using the Lowry method. After addition of SDS–PAGE sample buffer and boiling, 40 *μ*g of denatured proteins were separated in 12% SDS–PAGE and then transferred to nitrocellulose papers. After the blotting, nitrocellulose papers were incubated with specific antibodies. The primary antibodies used were: polyclonal anti-COX-2 (Cayman Chemicals, USA), anti-phospho-STAT-1(Tyr701) (Cell Signaling, USA) and anti-*β*-actin (Sigma, USA); monoclonal anti-COX-1 (Cayman Chemicals, USA) and anti-STAT-1 p84/p91 (Santa Cruz Biotechnology, USA). Secondary antibodies (HRP conjugated) were purchased from Santa Cruz Biotechnology, USA. Immunolabelling was visualised by using the ECL procedure (Amersham Biosciences, USA). Bands were quantified by using a densitometric image analysis software (Image Master VDS, Pharmacia Biotech, Uppsala, Sweden). Normalisation was made against *β*-actin expression.

### Determination of COX-2 activity

Conditioned media from HUVE cells were collected 48 h after transfection with siRNAs and stored at −80°C. Cyclooxygenase-2 enzymatic activity was evaluated by measuring the 6-keto-PGF1*α* release in conditioned media by using an ELISA assay (Assay Designs, USA), as previously described (Spisni *et al*, 2003). 6-keto-PGF1*α* production was then related to the protein concentration in cell lysates. PGE2 levels in HT29 culture media were evaluated by using an ELISA assay (Cayman Chemicals, USA) and related to the protein concentration in cell lysates.

### *In vitro* angiogenesis test

*In vitro* angiogenesis was evaluated by seeding HUVE cells on a 3-D collagen gel in a 24-well plate, as previously described (Spisni *et al*, 2003), and transfecting them with siRNAs. Briefly, collagen gel was prepared by adding eight volumes of collagen solution (3 mg ml^−1^, Roche Applied Science, USA) to two volumes of a mixture containing M199 5 × , HEPES 0.02 M and NaHCO_3_ 7.5 mg ml^−1^. After pH adjusting to 7.2–7.4, the mixture was quickly dispensed to the wells and gelification was achieved at 37°C. After gelification, wells were washed twice with M199. Human endothelial cells (5 × 10^4^ well^−1^) were seeded onto the gels and then transfected with siRNAs (final concentration 200 pM) by using Oligofectamine reagent (Invitrogen, USA) according to the manufacturer's instructions. *In vitro* capillary-like formation was stimulated with 40 nM PMA, examined 48 h after transfection by using a phase contrast microscope and the number of tubular capillary-like structures per well was counted.

### Cloning anti-COX-2 shRNA into pSUPER retroviral vector

**Figure 1:**
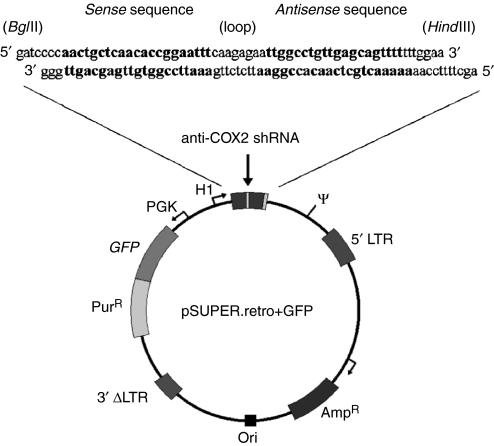
Scheme of pSUPER.retro vector. A specific sequence for anti-COX-2 short hairpin-RNA (sense sequence – loop – antisense sequence) was cloned into pSUPER.retro vector and the transcription of this sequence is regulated by H1 promoter for RNA pol-III. GFP gene expression provides a rapid test for infection efficiency and the gene for puromycin resistance is necessary to select clones expressing shRNAs against COX-2 mRNA. All cassettes are included into retroviral 5′–3′ LTRs to allow provirus integration in host cells genome. 3′ LTR is inactivated by deletion to avoid virus replication inside infected cells (see details under Materials And Methods).

Constructs, coding for anti-COX-2 shRNA, were prepared as described by Brummelkamp *et al* (2002a). pSUPER.retro vector (Oligoengine, Seattle, WA, USA), based on the murine stem cell virus (MSCV) genome, was a kind gift of Dr P Chieco (CRBA lab, Bologna, Italy). Forward and reverse sequences for anti-COX-2 shRNA construct were 5′-gatccccaactgctcaacaccggaatttcaagagaattccggtgttgagcagtttttttggaa-3′ and 5′-agcttttccaaaaaaactgctcaacaccggaattctcttgaaattccggtgttgagcagttggg-3′, respectively, as shown in Figure 1. 64 nt-containing oligos were synthesised and purchased from PROLIGO (USA). Sequence design started from the most effective anti-COX-2 synthetic siRNA (sequence B). Steps for cloning oligonucleotides into pSUPER.retro vector were made accordingly to pSUPER RNAi system protocol (www.oligoengine.com). Forward and reverse oligonucleotides were annealed to form a duplex. The annealed oligos were then ligated into the *Bgl*II–*Hind*III cleavage site within the pSUPER.retro vector prelinearised with the same restriction enzymes. Recombinant vector containing inserts was transformed into competent *Escherichia coli* cells. After selection in ampicillin-containing medium, colonies were recovered and checked for the presence of recombinant pSUPER.retro vector.

### Virus production and cell infections

Anti-COX-2 pSUPER.retro. vector was transfected into Phi-NX (‘Phoenix’) packaging cell line kindly provided by Dr P Chieco (CRBA lab, Bologna, Italy) to produce ecotropic retroviral supernatants. Phoenix cells were cultured in Dulbecco's modified Eagle's medium (DMEM) supplemented with 10% FCS and pretreated with Chloroquine at final concentration of 25 *μ*M. The day before transfection, Phoenix cells were seeded in 10 cm dishes (3 × 10^6^ cells dish^−1^) in order to reach 60% confluence at the time of transfection. Cells were transfected with 10 *μ*g of viral vector DNA by using calcium–phosphate precipitation method (Wigler *et al*, 1977; Chen and Okayama, 1987). At 48 h after transfection, culture medium was filtered through a 0.45 *μ*m filter and the viral supernatant was used for HT29 cells infection after addition of 8 *μ*g ml^−1^ of polybrene (Sigma, USA). After infection, HT29 cells were incubated at 32°C in 5% CO_2_ for 6 h. Then medium was changed with fresh medium and HT29 cells were allowed to recover for 48 h at 37°C in 5% CO_2_. Infection efficiency was examined under a fluorescence microscope to check the green fluorescent protein (GFP) expression. Infected cells were selected by adding puromycin (1 *μ*g ml^−1^) for 48 h to the culture medium. Cyclooxygenase-2 expression in HT29 wild-type cells and in HT29 pSUPER(+) clones (producing anti-COX-2 shRNA) was analysed by Western blot and by real-time polymerase chain reaction (RT-PCR), as described in this section. The same procedure was used to infect HUVE, HCA7 and HeLa cells and to obtain HT29 pSUPER(−) clones infected with empty vector, not expressing anti-COX-2 shRNA. Infection efficiencies were evaluated by using confocal microscopy analysis.

### Real-time polymerase chain reaction

Total RNA was purified starting from subconfluent HT29 cells (wild type and pSUPER(+)) by using Eurozol reagent (CELBIO, Milan, Italy) according to the manufacturer's instructions. Total RNA was quantified by spectrophotometry and analysed by electrophoresis on 1% agarose/formaldehyde denaturing gel to exclude the presence of RNA degradation. Extracted total RNA samples were then treated with DNase I, to remove any genomic DNA contamination, by using DNA-*free* kit (Ambion, USA). mRNA levels were analysed by real-time PCR by using a Bio-Rad iCycler system (Bio-Rad, USA) according to the manufacturer's instructions. Cyclooxygenase-2 mRNA expression was evaluated in both HT29 wild-type and HT29 pSUPER(+) cells. The specific primer pair for COX-2 was designed by using Beacon Designer 2.0 software. The homology with other human sequences and the formation of template secondary structures were carefully avoided. Cyclooxygenase-2 primers had the following sequences: 5′cctgtgcctgatgattgc3 (forward) and 5ctgatgcgtgaagtgctg3 (reverse). The primer pair for the housekeeping *β*-glucuronidase (GUSB) gene had the following sequences: 5′tggtataagaagtatcagaagcc3′ (forward) and 5′gtatctctctcgcaaaaggaac3′ (reverse). Cellular total RNA was reverse-transcripted into cDNAs and then amplified by using a SYBR supermix kit (Bio-Rad, USA) for 40 cycles at 95°C for 30 s, 53°C for 20 s and 72°C for 30 s. The melting curve data were collected to check PCR specificity. Each cDNA sample was analysed as triplicate and corresponding samples with no cDNAs were included as negative controls. Cyclooxygenase-2 mRNA levels for each sample were normalised against GUSB mRNA levels and relative expressions were calculated by using *C*_t_ values.

### MTT assay

HT29 cells (wild type, pSUPER(−) and pSUPER(+)) were seeded in 24-well plates (1 × 10^4^ cells well^−1^) and the MTT assay was performed in triplicate at days 2, 5, 8, 12, 15 and 20. Briefly, medium was replaced with fresh complete medium (450 *μ*l). A measure of 50 *μ*l of PBS containing 5 mg ml^−1^ MTT (Sigma, USA) was added to each well. In the absence of light, samples were incubated for 2 h and precipitates were resuspended by adding 100 *μ*l of 10% SDS solution to each well. Absorbance was measured spectrophotometrically on a plate reader (Bio-Rad, USA) at 570 nm.

### BrdU labelling index

The cell cycle distribution of HT29 cells (wild type, pSUPER(−) and pSUPER(+)) was evaluated by using a cytofluorimeter, as previously described (Landuzzi *et al*, 2000). Briefly, 1 × 10^6^ cells were seeded in complete medium. After 24 h from seeding, cell cultures were incubated with 10 μM BrdU (Sigma, USA) for 1 h in a CO_2_ atmosphere at 37°C. Harvested cells were fixed in 70% ethanol for 30 min. After DNA denaturation with 2 N HCl for 30 min at room temperature, cells were washed with 0.1 M Na_2_B_4_O_7_ (pH 8.5). Cells were then processed for indirect immunofluorescence staining, using *α*-BrdU (Becton Dickinson, Milan, Italy) diluted 1 : 4 as a primary MAb, and stained with 20 *μ*g ml^−1^ propidium iodide before flow cytometry analysis (FACSCalibur, Becton Dickinson, Milan, Italy).

### Cell migration assay

Migration assay was performed by using Boyden chambers (New Technologies Group, Italy) with 8-*μ*m pore polycarbonate membranes (New Technologies Group, Italy). Membranes were coated with Matrigel (Sigma, USA) at 40-fold dilution. Assay was performed using fresh DMEM supplemented with 10% heat-inactivated FCS as chemoattractant agent. HT29 cells (wild type, pSUPER(−) and pSUPER(+)) were added into the upper chamber at high density (500 × 10^3^ cells) either in the absence or presence of PMA 40 nM stimulation and then incubated for 24 h at 37°C. Following incubation, membranes were disassembled and nonmigratory cells on the upper surface of the membrane were wiped with a cotton swab. Cell invasion was determined by counting under light microscopy the number per optical fields (× 200 magnification) of the cells that migrated to the lower side of each membrane, after fixing and staining membranes with 2% Toluidine Blue.

### Soft-agar colony formation assay

Anchorage-independent growth was determined in 0.33% agarose (SeaPlaque, FMC BioProducts Rockland, ME, USA), as described by Benini *et al* (2004). HT29 cell suspensions (wild type, pSUPER(−) and pSUPER(+); 1000 cells sample^−1^) were plated in a semisolid medium (DMEM supplemented with 10% FCS and 0.5% agar). Dishes were incubated at 37°C in a humidified atmosphere containing 5% CO_2_ and colonies were counted (under light microscopy) after 7 days.

### Confocal microscopy and immunofluorescence analysis

To visualise the localisation of phospho-STAT-1 (Tyr701) protein, HUVE and HT29 cells were grown on cover slides, washed in TBS buffer, fixed with 4% paraformaldehyde, permeabilised with PBS-Triton X-100 0.1% and quenched with cold 0.1% sodium borohydride in TBS. Cells were treated with blocking buffer (PBS containing 10% horse serum and 1% BSA) and then incubated with phospho-STAT-1 (Tyr701) primary antibody diluted 1:100 in TBS-BSA 1%. After washings, cells were incubated with secondary anti-rabbit TRITC-conjugated antibody (Dako, Denmark) diluted 1:50 in TBS-BSA 1%. Finally, slides were washed and mounted in glycerol-PBS medium containing 50 mg ml^−1^ DABCO. To evaluate pSUPER.retro infection system efficiency, cells (HUVE, HT29, HCA7 and HeLa) were seeded on cover slides 24 h after infection, fixed with 4% paraformaldehyde and permeabilised with PBS-Triton X-100 0.1%. Nuclei were stained with propidium iodide 0.05 *μ*g ml^−1^ and slides were mounted, after washings, in glycerol-PBS medium containing 50 mg ml^−1^ DABCO. The imaging was performed on a confocal microscope (Leica, Germany) equipped with an argon/krypton laser. Optical sections were obtained at increments of 0.5 *μ*m in the *Z*-axis and were digitised with a scanning mode format of 512 × 512 pixels. The image processing and the volume rendering were performed using the Leica TCS software.

### Statistical analysis

Data were expressed as mean±s.e.m. Differences were analysed by Student's *t*-test and considered statistically significant at *P*<0.05 and *P*<0.01.

## RESULTS

**Figure 2:**
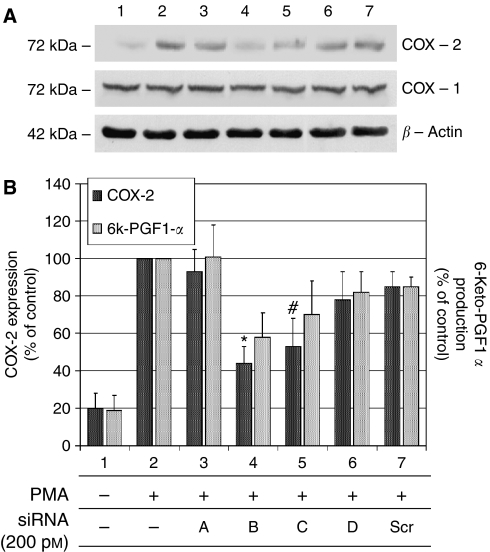
COX-2 specific knockdown by siRNAs in HUVE cells and evaluation of 6-keto-PGF1*α* production. HUVE cells were transiently transfected with siRNAs directed against COX-2 mRNA (sequences **A**–**D**; final concentration 200 pM): COX-2 levels (dark bars) and 6-keto-PGF1*α* production (bright bars) were analysed by Western blot and ELISA assay, respectively. All procedures are described under Materials and Methods. (**A**) Shows COX-2 and COX-1 expression after siRNAs treatment. After the evaluation of the bands intensity by Image Master VDS software, both COX-2 and COX-1 levels were normalised against *β*-actin expression. All samples (lanes 2–7) except control in lane 1 were treated with PMA 40 nM. Lanes 3–6: samples treated with siRNA A, B, C and D, respectively. Lane 7: HUVE cells transfected with siRNA-Scr (scrambled), representing a negative control. Data are expressed as % of PMA-stimulated control value (lane 2) and represent the mean±s.e.m. of three independent experiments. ^*^(*P*<0.01); #(*P*<0.05).

**Figure 3:**
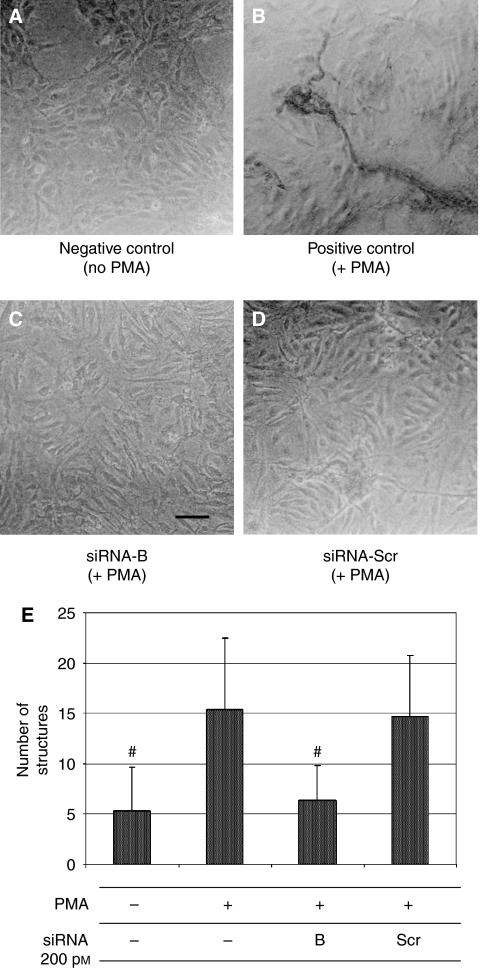
Small interfering RNA-B (siRNA-B) inhibition of PMA-induced angiogenesis on 3-D collagen gel. HUVE cells, seeded on 3-D collagen gels, were transfected with siRNA-B and siRNA-scrambled (**C** and **D** respectively; final concentration 200 pM) and treated for 48 h with PMA 40 nM in order to stimulate the early formation of capillary-like tubular structures. Results were compared with negative control (no PMA treatment, **A**) and PMA-stimulated positive control (**B**). The graph in (**E**) shows the number of capillary structures formed for each sample after treatments. All procedures are described under Material and Methods and results are expressed as mean±s.e.m. of three different experiments. #(*P*<0.05). Bar: 20 *μ*m.

**Figure 4:**
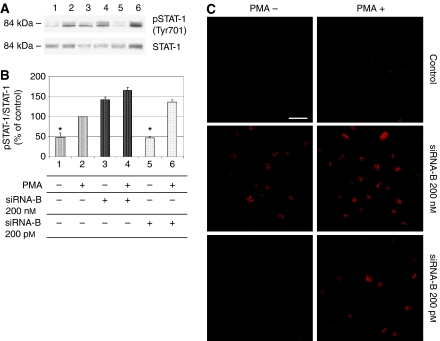
Small interfering RNA-B (siRNA-B) activates the interferon-signalling cascade in HUVEC only at high concentration. Cells were transiently transfected with siRNA-B directed against COX-2 mRNA as described under Material and Methods. Two different final concentrations were used (200 nM and 200 pM). Phospho-STAT-1 (Tyr701) and STAT-1 proteins expression was analysed by Western blot (**A**) and pSTAT-1 levels, normalised with respect to STAT-1 total levels, are reported in (**B**). Samples in lanes 2, 4 and 6 were treated with PMA 40 nM and represent positive controls. Lanes 1: negative control (no PMA stimulation). Lanes 3 and 4: samples treated with siRNA-B 200 nM. Lanes 5 and 6: samples treated with siRNA-B 200 pM. Data are expressed as % of positive control value in lane 2 and represent the mean±s.e.m. of three independent experiments. The same treatments were used in an immunofluorescence assay to determine the phospho-STAT-1 protein levels and localisation in siRNA-transfected HUVE cells (results are shown in **C**). ^*^(*P*<0.01). Bar: 20 *μ*m.

**Figure 5:**
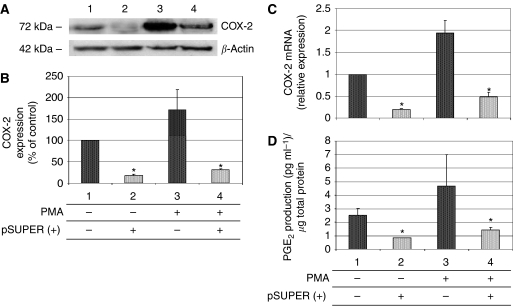
Stable knockdown of COX-2 gene by RNAi in HT29 cells. HT29 cells were infected using pSUPER.retro system, selected and analysed for COX-2 protein and COX-2 mRNA levels by Western blot and real-time PCR (**A**–**C**, respectively). PGE2 production (**D**) was evaluated by using an ELISA assay. All procedures are described under Material and Methods. Cyclooxygenase-2 expression in infected cells (lanes 2 and 4) was compared with that of control cells (lanes 1 and 3), in the absence (lanes 1–2) or in the presence (lanes 3–4) of 40 nM PMA-stimulation. Data from Western blot are expressed as % of PMA-stimulated control in lane 1. All results are expressed as mean±s.e.m. of three different experiments. ^*^(*P*<0.01).

**Figure 6:**
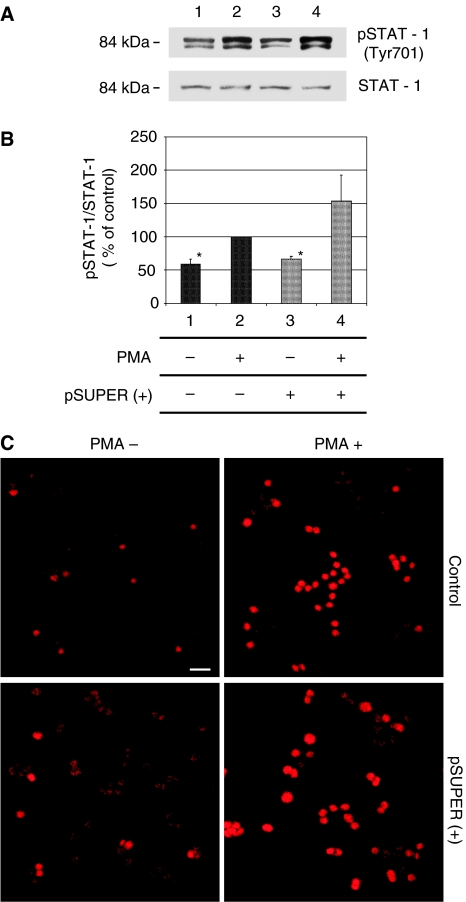
Effect of anti-COX-2 shRNA expression on the interferon-signalling cascade in HT29 pSUPER(+) cells. An analysis of STAT-1 phosphorylation in HT29 wild-type cells and HT29 cells infected using pSUPER.retro system was performed by Western blot (**A** and **B**) and immunofluorescence (**C**) assays, following the procedures described under Material and Methods. Phospho-STAT-1 (Tyr701) and STAT-1 proteins expression was analysed by Western blot (**A**) and pSTAT-1 levels, normalised with respect to STAT-1 total levels, are reported in (**B**). Lanes 1 and 2: HT29 wild type. Lanes 3 and 4: HT29 pSUPER(+). Samples in lanes 2 and 4 were treated with PMA 40 nM. Data are expressed as % of positive control value in lane 2 and represent the mean±s.e.m. of three independent experiments. The same samples were analysed in an immunofluorescence assay to determine the phospho-STAT-1 protein level and localisation (results are shown in **C**). ^*^(*P*<0.01). Bar: 20 *μ*m.

**Figure 7:**
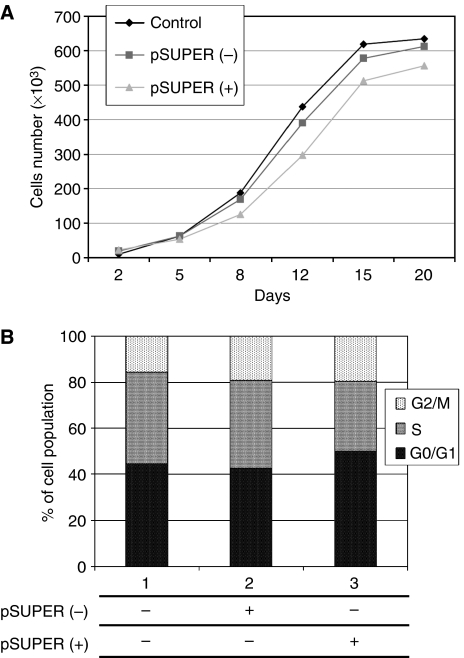
Effect of pSUPER.retro infection system on the viability and the cell cycle distribution of HT29 cells. Proliferation curves were determined by the MTT assay (**A**) and the cell cycle distribution analysis was carried out on 1 × 10^6^ cells samples^−1^ after 60 min of incubation with BrdU (**B**). All procedures are described under Material and Methods. Control: HT29 wild-type cells (• and lane 1); pSUPER(−): HT29 cells infected with vector nonexpressing anti-COX2 shRNA (▪ and lane 2); pSUPER(+): infected HT29 cells expressing shRNA against COX-2 mRNA (▴ and lane 3). All data represent the mean of three independent experiments.

**Figure 8:**
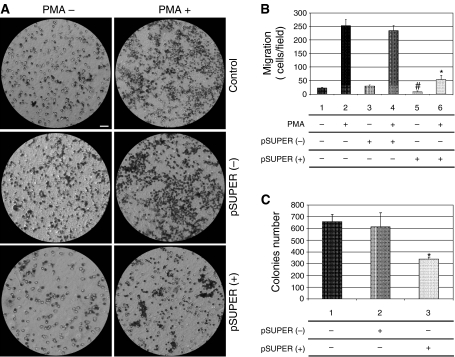
Effect of pSUPER.retro infection system on migration and soft-agar colony formation in HT29 cells. The migration assay was performed by using Boyden chambers and 8-*μ*m polycarbonate membranes coated with Matrigel (40-fold dilution). After 24 h of incubation, cells that migrated through the Matrigel-coated membranes were fixed, stained, photographed (**A**) and counted under light microscopy (**B**). Regarding the soft-agar colony formation assay, the number of colonies was evaluated 7 days after the seeding in soft agar (**C**). Control: HT29 wild-type cells; pSUPER(−): HT29 cells infected with vector nonexpressing anti-COX2 shRNA; pSUPER(+): infected HT29 cells expressing shRNA against COX-2 mRNA. In the migration assay samples were tested in the absence and presence of PMA 40 nM. All procedures are described under Material and Methods and data in (**B**) and (**C**) represent the mean±s.e.m. of three independent experiments. ^*^(*P*<0.01); #(*P*<0.05). Bar: 20 *μ*m.

**Figure 9:**
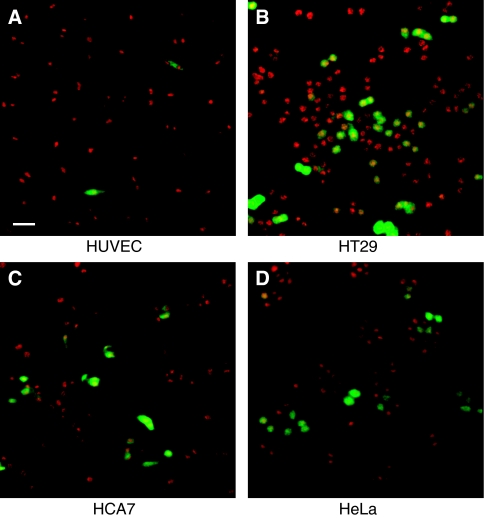
Efficiency of pSUPER.retro infection system. The images represent four different cell types that underwent the infection by pSUPER.retro system (**A**: HUVEC; **B**: HT29; **C**: HCA7; **D**: HeLa). Cells were fixed 24 h after infection and observed by using confocal microscopy. Nuclei were stained with propidium iodide and the infected cells appear GFP-positive since the pSUPER.retro vector contains an expressing cassette for GFP gene. Bar: 20 *μ*m.

Considering the relevance of endothelial COX-2 in the angiogenic process, we used an *in vitro* angiogenesis experimental model, based on primary HUVEC, to detect whether siRNA molecules were capable of downregulating COX-2 expression and inhibiting COX-2-dependent angiogenesis. Four different siRNAs, directed against COX-2 mRNA, were transfected at 200 pM concentration, by using the Oligofectamine reagent, in HUVEC treated with PMA to enhance COX-2 expression. As shown in Figure 2, only two siRNAs (sequences B and C) were capable of reducing COX-2 protein levels by more than 50%, whereas a scrambled siRNA, used as a negative control, was found to be completely devoid of effects. Moreover, we demonstrated that the transient knockdown mediated by siRNAs in HUVEC was highly specific since COX-1 expression resulted unaffected (Figure 2A). In samples in which COX-2 was downregulated, also PGI2 production, evaluated by ELISA assay, significantly decreased up to more than 40% (Figure 2B). Thus, we chose siRNA sequence-B to perform an *in vitro* angiogenesis test (Figure 3). As reported in the literature (Ilan *et al*, 1998), HUVE cells were able to organise into capillary-like tubular structures when seeded on 3-D collagen gel and stimulated with PMA (compare PMA-stimulated cells in Figure 3B to control cells in A). We observed that transfection of siRNA-B in HUVEC strongly affected their ability to organise in tubular structures (Figure 3C), with a significant reduction of vessels number after PMA stimulation (as shown in Figure 3E). Cells transfected with scrambled siRNA (Figure 3D) were still able to differentiate in tubular structures with the same efficiency of PMA-stimulated control cells (as shown in Figure 3E), allowing to exclude toxicity and nonspecific effects of siRNA-B on angiogenesis. These results demonstrate that siRNAs are capable to affect the *in vitro* angiogenic process by downregulating COX-2 expression in a strong specific manner. We also evaluated whether the transfection of synthetic siRNAs in HUVE cells may activate the interferon-mediated Jak-STAT pathway, as previously reported for other siRNAs molecules (Sledz *et al*, 2003). Western blot analysis of phospho-STAT-1(Tyr701) (active form) levels, normalised against p85/p91 STAT-1 total protein levels, showed that only an high concentration (200 nM) of transfected siRNA-B is able to trigger the interferon system response, whereas a lower but effective dose of siRNA (200 pM) does not have any effect on STAT-1 phosphorylation (Figure 4A and B). Phorbol 12-myristate 13-acetate-treated samples were used as positive controls, as suggested by the literature (Cohen *et al*, 2005). Data from immunofluorescence analysis were in high agreement with these findings (Figure 4C). STAT-1 phosphorylation, followed by nuclear translocation, was strongly increased in samples transfected with siRNA 200 nM, while no relevant differences were detected between samples transfected with siRNA 200 pM and controls. In order to achieve a stable downregulation of COX-2 in cancer cells, we prepared a mammalian vector expressing an anti-COX-2 shRNA. We used the pSUPER.retro vector system, capable of integrating expression cassettes in human genome, to produce efficient and specific downregulation of COX-2 gene expression induced by a siRNA mechanism (Brummelkamp *et al*, 2002a, 2002b). We cloned the dsRNA sequence corresponding to siRNA-B into a pSUPER.retro vector containing also the expression cassette for the GFP. The recombinant vector was transfected into Phoenix packaging cells to produce retroviral ecotropic supernatant, used to infect HT29 cells. Infected cells were selected by using standard puromycin treatment (1 *μ*g ml^−1^) for 48 h. Selected HT29 cells (HT29 pSUPER(+)) were analysed by Western blot for COX-2 expression. As shown in Figure 5(A and B), COX-2 levels were found to be significantly decreased (more than 70%) in HT29 pSUPER(+) when compared to control cells. The inhibition was still effective when the COX-2 gene expression was stimulated by PMA treatment. Cyclooxygenase-2 mRNA levels were analysed in HT29 pSUPER(+) by real-time PCR. Results were in strict accordance with data obtained by Western blot, confirming the specific COX-2 mRNA degradation by RNAi. In fact, we obtained an 80% reduction of COX-2 mRNA levels either in the absence or in the presence of PMA stimulation (Figure 5C). As a further demonstration of the efficiency of the COX-2 knockdown mediated by RNAi, we also found a significant decrease of PGE2 production in HT29 pSUPER(+) cells (Figure 5D). Since we found, as mentioned above, that the transfection of exogenous synthetic siRNAs is capable to activate the interferon system at high concentrations in HUVE cells, the following aim was to demonstrate whether an endogenous and constitutive production of shRNA in the HT29 pSUPER(+) model had a different effect. Surprisingly, we found that shRNAs, that strongly downregulate COX-2 expression in HT29 pSUPER(+) cells, did not trigger the interferon system response in the absence of PMA treatment, compared to the control. Both Western blot (Figure 6A and B) and immunofluorescence (Figure 6C) analysis of phospho-STAT-1(Tyr701) levels and localisation confirmed this evidence. Moreover, in order to obtain more data on the effects of the constitutive COX-2 downregulation in HT29 pSUPER(+) cells, we performed four different assays to evaluate the proliferation profile and the invasiveness of these clones, compared to two different controls: HT29 wild-type and HT29 pSUPER(−) cells. HT29 pSUPER(−) cells were selected with puromycin after infection with the retroviral vector, devoid of the anti-COX-2 shRNA expression cassette. Although the MTT proliferation assay (Figure 7A) and the cell cycle distribution analysis (Figure 7B) did not show significant differences between controls and HT29 pSUPER(+), data from migration assay performed with Boyden chambers (Figure 8A and B) and soft-agar colony formation assay (Figure 8C) suggest that the stable knockdown of COX-2 gene by RNAi promotes a significant reduction of the migratory ability as well as a strong inhibition of colony formation in soft agar in infected pSUPER(+) colon cancer cells. Interestingly, the loss of the malignant behaviour *in vitro* of pSUPER(+) HT29 cells did not seem to depend on an impairment of cell growth, since constitutive expression of anti-COX-2 shRNA in HT29 cells only slightly modified their proliferation profile and their cell cycle distribution, but it derived from a reduction of the ability to invade the extracellular matrix and to grow in anchorage-independent manner, which are indexes of an invasive and aggressive behaviour. In the light of future possible *in vivo* applications, we finally tested the efficiency of the pSUPER.retro infection system on HUVEC and different cancer cell lines. The infection efficiency on HUVE cells was very low (less than 5%), even if repeated attempts were performed. In contrast, HT29 and HCA7 colon cancer cell lines, compared to HeLa cells (used as positive control), were easily infected showing higher efficiency levels (Figure 9). The infection efficiency for both HT29 and HCA7 was around 45%, whereas it was around 35% for HeLa cells.

## DISCUSSION

RNAi represents a brand new approach in the field of reverse genetics, since it is a potent tool capable of silencing genes in a high, long lasting and selective manner. The understanding of RNAi mechanism of action has soon disclosed a wide spectrum of possible applications, either *in vitro* or *in vivo*. In the past decade, many studies have shown that small dsRNA molecules are capable of downregulating genes with sequence homology and that the knockdown mediated by RNAi machinery is an ubiquitous phenomenon in eukaryotic cells. Much of the knowledge regarding RNAi comes from studies on *C. elegans*. In this organism, Mello and co-workers in 1998 demonstrated that microRNAs (miRNAs) physiologically regulate the expression of genes involved in worm development (Fire *et al*, 1998; Tuschl *et al*, 1999; Elbashir *et al*, 2001b; Tijsterman *et al*, 2002). Today, siRNAs and shRNAs are widely used by researchers to silence the expression of many target genes, because of their high specificity and their apparent nontoxicity. Moreover, systems based on DNA plasmids or retroviral vectors have provided new solutions to achieve a stable knockdown mediated by shRNAs.

In this work, we used a RNAi-based approach to obtain an efficient knockdown of COX-2 mRNA. The overexpression of COX-2 seems to play a critical role in many pathological processes. In particular, the concept that high levels of PGI2 and PGE2, the main products of arachidonate metabolism in vascular tissues and cancer cells, respectively, stimulate both tumour-induced angiogenesis and tumour progression is widely accepted (Leahy *et al*, 2002; Iñiguez *et al*, 2003), whereas the overexpression of the inducible form of COX enzyme in colorectal cancer cells seems to promote tumour invasion, tumour growth and tumour metastasis (Gupta and Dubois, 2001; Yamauchi *et al*, 2002; Romano and Claria, 2003; Tuynman *et al*, 2004). Moreover, the possibility that the overexpression of genes implicated in human cancers may be due to a downregulation or a general imbalance of specific miRNAs has been recently supported (Michael *et al*, 2003; Calin *et al*, 2004; He *et al*, 2005; Lu *et al*, 2005) and it could be investigated in the case of colorectal cancer.

In our experiments, we tested the effect of the transfection of four different sequences of anti-COX-2 siRNAs on HUVE cells, stimulated with PMA in order to overexpress COX-2 mRNA. It has been previously shown that PMA stimulates COX-2 expression in HUVEC, strongly increasing both mRNA and protein levels (Hirai *et al*, 1999). Under these conditions, the release of prostacyclin (PGI2) was also found to be highly augmented. It is well known that PGI2 stimulates angiogenesis, probably by acting on a nuclear receptor belonging to peroxisome proliferator-activated receptors (PPARs) family (Spisni *et al*, 2001; Pola *et al*, 2004). Two of the synthetic siRNAs tested (sequences B and C) resulted to be effective in downregulating COX-2 levels and enzymatic activity in HUVE cells in a specific manner, having no effect on COX-1 expression. Moreover, COX-2 silencing by siRNAs was observed even at very low concentrations (200 pM). Has to be mentioned that one of these active siRNA sequences (sequence B) overlaps the same sequence used by Denkert *et al* (2003) to efficiently downregulate COX-2 expression in ovarian carcinoma cells. As a consequence of COX-2 downregulation and PGI2 reduction, endothelial cells transfected with siRNA-B failed to organise capillary-like tubular structures in 3-D collagen gel when stimulated with PMA, whereas control cells (nontransfected or transfected with a scrambled siRNA) rapidly formed several lengthened structures sprouting inside the gel.

It has been reported that synthetic siRNAs (Sledz *et al*, 2003; Kim *et al*, 2004; Judge *et al*, 2005), as well as short single-stranded RNAs (ssRNAs) (Kim *et al*, 2004) and some DNA vectors expressing shRNAs (Bridge *et al*, 2003), are able to trigger an interferon response *in vitro*. On the contrary, other findings demonstrate that is even possible to administer naked, synthetic siRNA to mice without inducing any interferon response (Heidel *et al*, 2004). In our experimental model, we analysed the phosphorylation status (on Tyr701) of STAT-1 protein as a marker of the interferon system activation (Brierley and Fish, 2005). As STAT-1 activation can be triggered by PMA treatment (Cohen *et al*, 2005), we compared the levels of STAT-1 phosphorylated isoform in HUVEC transfected with siRNA-B at two different doses (200 nM and 200 pM) with respect to PMA-stimulated control cells. While the treatment with a 200 nM dose of siRNA-B significantly increased STAT-1 phosphorylation levels, both in the absence or in the presence of PMA, the treatment with the same siRNA molecule at a low dose (200 pM) did not show any significant effect on STAT-1 phosphorylation when compared with controls. Similar results were obtained by analysing phospho-STAT-1 expression and its nuclear translocation by immunofluorescence assay, indicating that low but effective doses of anti-COX-2 siRNA are devoid of effects on the interferon system.

Following the detection of a siRNA sequence able to efficiently interfere with COX-2 expression in HUVE cells, we focused our attention on a new RNAi strategy based on the use of a shRNA-expressing vector. Our aim was to obtain a stable and efficient knockdown of COX-2 gene in HT29 cells, a cell line derived from a human colorectal cancer and known to overexpress this enzyme. The approach we chose was based on pSUPER.retro technology (Brummelkamp *et al*, 2002a, 2002b). Many advantages came from this approach, first of all the possibility to achieve, by using inexpensive tools, a potent silencing of target genes that is also highly specific and long lasting. Therefore, interesting results were achieved, since data from Western blot, real-time PCR analysis and ELISA assay clearly showed a strong and selective reduction of COX-2 protein and mRNA levels, with consequent inhibition of PGE2 production, in HT29 cells infected with a pSUPER.retro vector expressing anti-COX-2 siRNA-B (HT29 pSUPER(+)), even in the presence of PMA-induced COX-2 overexpression. These data support the evidence of a strong and long-lasting COX-2 mRNA degradation driven by siRNA molecules processed from shRNA precursors.

As previously tested in HUVEC treated with anti-COX-2 siRNA-B, we analysed the effect of the permanent expression of shRNA molecules on STAT-1 activation in HT29 pSUPER(+) cells, detecting no significant increase neither in STAT-1 phosphorylation levels nor in phospho-STAT-1 nuclear accumulation. These data suggest that a vector-based stable expression of shRNA molecules induces a weaker interferon system response with respect to the transfection of synthetic siRNAs.

Performing experiments aimed to analyse the phenotype of COX-2-deficient HT29 pSUPER(+) cells, we detected no significant effects of COX-2 downregulation on cell proliferation and cell cycle distribution, observing only a slight decrease of the proliferation rate and a slight accumulation of cells in the G0/G1 phase. These results are in line with other data recently collected in a model of ovarian carcinoma cells (Denkert *et al*, 2003) and in a model of human hepatocellular carcinoma cells (Park *et al*, 2005) based on siRNA-mediated COX-2 downregulation, suggesting that COX-2 itself is not decisively involved in the proliferation of human cancer cells as well.

However, we obtained interesting results by testing the *in vitro* invasive behaviour of HT29 pSUPER(+) cells and their anchorage-independent growth ability. Phorbol 12-myristate 13-acetate-stimulated HT29 cells, expressing high levels of COX-2, resulted to be able to degrade and migrate through ECM components of Matrigel-coated membranes, which indicates their malignant behaviour *in vitro,* associated with a well-described invasive and metastatic ability *in vivo* (Chen *et al*, 2001; Kakiuchi *et al*, 2002). Moreover, HT29 wild-type cells easily formed colonies in soft agar, which is an accepted criterion for transformation (Aaronson and Todaro, 1968) and an experimental condition that better represents tumour cells growth and invasiveness *in vivo* (Lawlor *et al*, 2002). The knockdown of COX-2 enzyme in HT29 cells abrogated their ability to invade Matrigel-coated membranes in a Boyden chamber assay, either in the absence or in the presence of PMA-stimulation, and strongly impaired their anchorage-independent growth in soft-agar basal conditions. These results support the involvement of COX-2 in the malignant behaviour of human colon carcinoma cells and underline the relevance of a stable virus-based COX-2 knockdown mediated by RNAi in order to impair the invasive and metastatic ability of CRC.

Our study confirms the predominant role that RNAi is assuming in the field of gene silencing owing to its high efficacy, specificity and nontoxicity. One of the novel targets in cancer therapies is to obtain a selective downregulation of those genes overexpressed in tumour tissues. Cyclooxygenase-2 is certainly one of them. Results reported here indicate an easy-to-use, powerful and high selective virus-based method to knockdown COX-2 gene in a stable and long-lasting manner, in colon cancer cells. Furthermore, they open up the possibility of an *in vivo* application of this anti-COX-2 retroviral vector, as therapeutic agent for human cancers overexpressing COX-2. In fact, we observed a significant resistance of HUVE cells to the pSUPER.retro viral infection in comparison with different types of human cancer cells, either CRC cells (HT29 and HCA7) and cervix carcinoma cells (HeLa). This observation indicates that endothelial cells are refractory to retroviral infection and suggests that this kind of virus-based approach might not affect the physiological prostaglandins production in vascular tissues, avoiding some of the well-known side effects coming from therapies based on selective COX-2 inhibitors (coxibs) (Ray *et al*, 2002; Fitzegerald, 2004; Horton, 2004; Mamdani *et al*, 2004; Oberholzer and Inamdar, 2004; Mitchell and Warner, 2005).
